# Oversensing of the wearable cardioverter defibrillator during bipolar ventricular stimulation

**DOI:** 10.1007/s00508-017-1290-z

**Published:** 2017-11-09

**Authors:** Martin Manninger, Tanja Odeneg, Friedrich Fruhwald, Helmut Brussee, Daniel Scherr

**Affiliations:** Division of Cardiology, Department of Medicine, Medical University of Graz, Auenbruggerplatz 15, 8036 Graz, Austria

**Keywords:** Pacing, Myocarditis, False alarm, Misdetection, Telemedicine

## Abstract

The wearable cardioverter defibrillator (WCD) is a temporary treatment option for patients with potentially reversible risk of sudden cardiac death. This case demonstrates a pitfall during WCD usage in a pacemaker-dependent patient as well as a possible solution allowing continuation of WCD therapy. Bipolar stimulation may lead to double counting of the WCD detection algorithm resulting in false alarm or inappropriate therapy.

## Case report

A 52-year-old man was referred to our institution with progressive dyspoea on exertion (New York Health Association NYHA class III). Echocardiography revealed severely depressed ejection fraction (EF) of 16% as well as severe left ventricular dilatation (left ventricular end diastolic diameter of 70 mm). The family history was negative for cardiovascular diseases, sudden cardiac death and syncope. The patient did not take any medications before admission. A dual-chamber pacemaker (Ensura MRI™ Surescan® Pacemaker, Medtronic, Dublin, Ireland) was implanted 3 months prior to admission due to third degree atrioventricular (AV) block of unknown cause which resulted in syncope. During follow-up, the patient was pacemaker-dependent (right ventricular pacing 100%, no intrinsic QRS complexes at an intervention rate of 30/min) and since echocardiography had shown a newly detected severely reduced ejection fraction, the patient was referred to our institution for coronary angiography and endomyocardial biopsy.

**Fig. 1:**
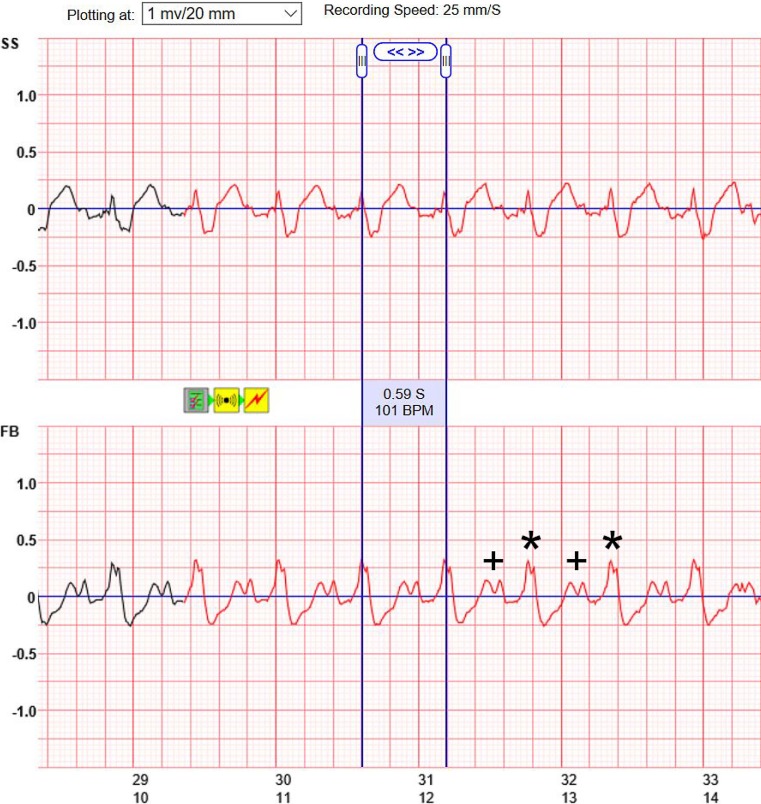
The recorded tracing on LifeVest Network (Zoll Medical, Chelmsford, MA, USA) shows sinus tachycardia at 101 bpm with PM activity in VAT mode. Red tracings indicate a detected VT event leading to an alarm (yellow icons between two channels side-side SS and front-back FB). *Blue lines* indicate the measures cycle length of 0.59 seconds (S) equivalent to a heart rate of 101 beats per minute (BPM). *P*-waves are indicated by a *plus (+)* and paced QRS complexes with asterisks (*), both were counted as ventricular activity, leading to a sensed heart rate of 202/min. Time of the recording in seconds is plotted on the x-axis, signal amplitudes on the y-axis. Recording speed is 25 mm/s, amplitudes are plotted at 1 mV/10 mm

Coronary artery disease was ruled out by angiography. Right ventricular endomyocardial biopsy was performed in the same session. Treatment with beta blockers, angiotensin-converting enzyme (ACE) inhibitors and spironolactone was initiated. Histologic work-up of the biopsies showed acute myocarditis with cardiomyocyte necrosis and suspected giant cell myocarditis (positive gene profile) plus myocardial infection with human herpesvirus type 6 (HHV-6 ribonucleic acid assay). As soon as the biopsy results were available, immune suppression with azathioprine, cyclosporine, methylprednisolone as well as antiviral therapy with valaciclovir were commenced. Due to the possible reversible cause of myocarditis, the patient received a WCD (LifeVest®, Zoll Medical, Chelmsford, MA, USA). The WCD’s ventricular tachycardia (VT) detection zone was programmed to 150 bpm, while the ventricular fibrillation (VF) detection zone was programmed to 220 bpm. Within the first 24 h of wearing the WCD, the device alarm went off 10 times after detecting VT/VF events. Recordings from the LifeVest Network showed oversensing of sinus tachycardia with large *P* waves and broad QRS complexes due to ventricular stimulation between 90 and 110 bpm during all alarm episodes (VAT mode, bipolar pacing at 3.0 V/0.4 ms at a threshold of 0.375 V/0.4 ms) (Fig. 1). Pacemaker interrogation showed a mean heart rate of 80 bpm and episodes of sinus tachycardia at maximum tracking rate of 130 bpm. During these episodes, tracings from LifeVest Network showed false alarms due to double counting during ventricular pacing by the WCD. The patient adequately terminated all alarms by pressing the response button and no inappropriate therapy was delivered.

During the following days, mean heart rate, peak heart rates as well as WCD alarms reduced after up-titration of beta blocker therapy (bisoprolol 2 × 5 mg) and addition and up-titration of ivabradine (2 × 7.5 mg). The WCD was programmed to a VT detection zone at 200 bpm and the VF detection zone to 250 bpm before the patient was discharged from hospital 2 weeks after admission with an EF of 20% and in NYHA class II.

During follow-up, the patient had significantly less false alarms due to oversensing and no sustained VTs. The pacemaker was regularly interrogated for episodes of VT below the WCD’s programmed VT detection zone. The patient is followed up regularly for levels of cyclosporine A and WCD treatment is planned for 6 months before deciding on upgrading to a cardiac resynchronisation therapy device with defibrillator (CRT-D).

## Discussion

Treatment with a WCD may be considered in patients with poor ejection fraction at high risk of sudden cardiac death who are currently not candidates for an implantable cardioverter defibrillator (ICD; i. e. bridge to transplantation, peripartum cardiomyopathy, active myocarditis or postmyocardial infarction; [1]). The prospective WEARIT II registry enrolled 2000 patients with postmyocardial infarction, postpercutaneous coronary intervention/coronary artery bypass graft or with non-ischemic cardiomyopathy and showed a VT/VF incidence of 1.1% during a median wearing duration of 90 days, which were all successfully terminated by the WCD. Inappropriate shock rate was 0.5% but no episodes of oversensing during pacemaker stimulation have been reported [2].

The LifeVest uses four electrodes to record two surface leads (front-back and side-side). The obtained bipolar recordings are analyzed for the presence of VT or VF using the TruVector DSP™ algorithm. This algorithm automatically discards one of the leads in case of artefacts or clipping. Heart rate is calculated by QRS detection and analysis of a Fournier transformation frequency plot. Additionally, QRS morphology is analyzed as a vectorcardiogram, which is compared with a template recorded at baseline. Similar to ICDs, the algorithm also uses stability (use of heart rate stability to discriminate between supraventricular tachycardias [SVTs] and VTs) and onset (use of sudden arrhythmia onset to discriminate between sinus tachycardia, SVTs and VTs) criteria [3]. In this case, the proximity of the end of the *T* wave and the *P* wave during VAT pacing resulted in double counting resulting in false alarms, which might have been caused by template mismatching. Although misdetection of the LifeVest’s algorithm has been reported during unipolar pacing, this is the first report of double counting during bipolar ventricular stimulation [3].

This case shows an important limitation of the WCD, since the device allows no re-programming in sensing thresholds or blanking periods by the treating physician. Multiple false alarms could lead to reduced compliance and early termination of WCD treatment by the patient at risk of sudden cardiac death. In addition, misdetection may lead to inappropriate shocks by the device. Special attention has to be paid to the heart rate profile in patients with implanted pacemakers who receive a WCD regardless of stimulation polarity. In the case presented here, strict heart rate control resolved further misdetections and prevented early termination of the WCD treatment by the patient.

## References

[1] Priori SG, Blomstrom-Lundqvist C, Mazzanti A, Blom N, Borggrefe M, Camm J (2015). 2015 ESC Guidelines for the management of patients with ventricular arrhythmias and the prevention of sudden cardiac death: The Task Force for the Management of Patients with Ventricular Arrhythmias and the Prevention of Sudden Cardiac Death of the European Society of Cardiology (ESC). Endorsed by: Association for European Paediatric and Congenital Cardiology (AEPC). Eur Heart J.

[2] Kutyifa V, Moss AJ, Klein H, Biton Y, McNitt S, MacKecknie B (2015). Use of the wearable cardioverter defibrillator in high-risk cardiac patients: data from the Prospective Registry of Patients Using the Wearable Cardioverter Defibrillator (WEARIT-II Registry). Circulation.

[3] LaPage MJ, Canter CE, Rhee EK (2008). A fatal device-device interaction between a wearable automated defibrillator and a unipolar ventricular pacemaker. Pacing Clin Electrophysiol.

